# Development and Optimization of Multi-Well Colorimetric Assays for Growth of *Coccidioides posadasii* Spherules and Their Application in Large-Scale Screening

**DOI:** 10.3390/jof11100733

**Published:** 2025-10-11

**Authors:** Augusto Vazquez-Rodriguez, Jieh-Juen Yu, Chiung-Yu Hung, Jose L. Lopez-Ribot

**Affiliations:** Department of Molecular Microbiology and Immunology and South Texas Center for Emerging Infectious Diseases, The University of Texas at San Antonio, San Antonio, TX 78249, USA; jesus.vazquezrodriguez@utsa.edu (A.V.-R.); jiehjuen.yu@utsa.edu (J.-J.Y.); chiungyu.hung@utsa.edu (C.-Y.H.)

**Keywords:** *Coccidioides*, coccidioidomycosis, antifungal drugs, high throughput screening

## Abstract

*Coccidioides immitis* and *Coccidioides posadasii*, the causative agents of coccidioidomycosis, represent a major public health concern in endemic regions of North and South America. The disease spectrum ranges from mild respiratory illness to severe disseminated infections, with thousands of cases reported annually in the United States and an increasing recognition of its global impact. Despite existing antifungal therapies, treatment remains challenging due to toxicity, drug resistance, and limited therapeutic options. High-throughput screening platforms have revolutionized drug discovery for infectious diseases; however, progress in antifungal screening for *Coccidioides* spp. has been hampered by the requirement for Biosafety Level 3 (BSL-3) containment. To overcome these barriers, we leveraged an attenuated *C. posadasii* strain that can be safely handled under BSL-2 conditions. Here, we describe the development and optimization of 96-well and 384-well plate screening methodologies, providing a safer and more efficient platform for antifungal discovery. This approach enhances the feasibility of large-scale screening efforts and may facilitate the identification of novel therapeutics for coccidioidomycosis.

## 1. Introduction

Invasive fungal infections represent a persistent and growing threat to global public health, particularly among immunocompromised populations, where they are associated with significant morbidity and mortality. Among the most critical fungal pathogens are *Coccidioides immitis* and *Coccidioides posadasii*, the dimorphic fungi responsible for coccidioidomycosis, also known as “Valley fever”. These fungi are endemic to arid regions of North and South America, where they thrive in soil and are transmitted to humans via inhalation of airborne arthroconidia [1,2,3].

Coccidioidomycosis manifests as a spectrum of diseases, ranging from acute or chronic pulmonary infections to severe disseminated forms that can affect the central nervous system, blood, bones, and other organs [4,5,6,7]. The burden of coccidioidomycosis is substantial, with the Centers for Disease Control and Prevention (CDC) reporting 10,000 to 40,000 cases annually in the United States alone, predominantly in Arizona and California [8]. Alarmingly, coccidioidomycosis was associated with approximately 200 deaths per year from 1999 to 2021 [9]. The global significance of *Coccidioides* spp. as a public health threat was further emphasized in 2022 when the World Health Organization Antimicrobial Resistance Division included these pathogens in its First Fungal Priority Pathogens List (WHO FPPL) [10]. Despite advances in antifungal therapies, treatment options for coccidioidomycosis remain limited, often requiring prolonged courses of antifungal agents such as azoles and Amphotericin B, which are associated with toxicity, drug interactions, and the potential for resistance development [11,12,13]. This situation underscores the need for novel therapeutic agents with improved safety and potency profiles.

Large-scale screening techniques have revolutionized drug discovery by enabling the rapid and systematic evaluation of chemical libraries in search of bioactive compounds [14,15]. These platforms have proven valuable in identifying novel therapeutic agents, including both synthetic and natural products, against various pathogenic microorganisms [16,17,18,19,20]. However, the use of antimicrobial screening for *Coccidioides* spp. is inherently limited due to the requirement for Biosafety Level 3 (BSL-3) facilities, which introduce regulatory, logistical, operational, and cost barriers [21,22]. As a consequence, few studies have explored or implemented screening strategies for coccidioidomycosis, and existing methods are cumbersome and low-throughput. To address these challenges, we have taken advantage of the availability of an attenuated *C. posadasii* mutant strain *∆cts2/∆ard1/∆cts3* (*∆T*), lacking expression of two chitinase genes and one D-arabinotol-2-dehydrogenase that can be handled under BSL-2 conditions [23]. Here, we report on the development and optimization of robust 96-well and 384-well plate screening methodologies facilitating safer, more efficient antifungal discovery for coccidioidomycosis and potentially other high-containment pathogens.

## 2. Materials and Methods

### 2.1. Fungal Cultures

The attenuated mutant strain *C. posadasii* C735 *∆cts2/∆ard1/∆cts3 *(*∆T*) [23], maintained under biosafety level 2 (BSL-2) conditions, was used for all experiments in this study. Arthroconidia were propagated on glucose yeast extract (GYE) agar plates (1% glucose, 0.5% yeast extract, 1.5% agar) and incubated at 30 °C for 3 weeks. Arthroconidia were harvested by scraping the surface of the plates and homogenized via bead beating in phosphate-buffered saline (PBS). Hyphal fragments were removed by filtration through a 40 μm cell strainer, followed by washing and concentration of arthroconidia through centrifugation at 2000 rpm for 5–10 min. The final pellet was resuspended in PBS, and cell concentration was quantified using a disposable hemocytometer. The viability of the arthroconidia and CFUs was validated by plating on GYE plates. To induce the generation of initial spherules in vitro, we inoculated Converse media at a density of 1–3 × 10^8^ spores per 100 mL. Converse media is a chemically defined medium containing high concentration of different salts with glucose as the carbon source, and Tamnol-N for enhancing the release of endospores and reducing the aggregation of spherules [24]. Cultures were maintained in vented polypropylene flasks at 39 °C, 10% CO_2_, and incubated at 180 rpm for 24 h for arthroconidia to grow into spherules (20–40 µM in diameter). Cultures were centrifuged at 2000 rpm for 5–10 min to recover spherules and filtered through a 40-μm cell strainer to remove residual debris. Spherules were resuspended in RPMI-1640 medium without phenol red buffered with 2.0 g/L sodium bicarbonate (RPMI) (Gibco, Thermo Fisher Scientific, Waltham, MA, USA) and adjusted to the desired concentration based on experimental conditions.

### 2.2. Compounds

Niclosamide (NIC), Niclosamide ethanolamine (NEN), Hexachlorophene (HEX), and Amphotericin B (AmB) were procured from Sigma Aldrich (St. Louis, MO, USA). Antifungal solutions were prepared at 1 mM in dimethyl sulfoxide (DMSO), except for AmB, which was prepared as a stock solution at 250 µg/mL.

### 2.3. Growth of C. posadasii in 96-Well Plates for Assay Optimization and Adaptation for Drug Screening

Spherule initials were prepared as previously described and adjusted to the desired working inoculum in RPMI medium. Two 96-well plate (Corning, New York, NY, USA) layouts were utilized: a positive control plate containing AmB at 10 µg/mL in all wells and a negative control plate containing DMSO at a final concentration of 1% (*v*/*v*). Each well of the control plates was seeded with the working inoculum. Plates were then incubated at 39 °C with 10% CO_2_ for 24 h. At the endpoint, 100 µL of 2,3-bis (2-methoxy-4-nitro-5-sulfo-phenyl)-2H-tetrazolium-5-carboxanilide (XTT) (Thermo Fisher Scientific) solution (0.5 mg/mL, supplemented with 40 µM menadione) was added to each well, and the control plates were further incubated under the same conditions. XTT reduction was monitored at different time points by measuring absorbance at 490 nm (OD_490_) using a microtiter plate reader (Synergy H1, Agilent BioTek, Santa Clara, CA, USA). Raw absorbance readings from the positive and negative control plates were used to calculate the Z′ score statistics as previously described [25,26]. The Z′ (Z-prime) score is a statistical parameter used to assess the quality and robustness of high-throughput screening (HTS) assays. By considering both the dynamic range between positive and negative controls and their variability, it provides a measure of assay reliability. Z′ values above 0.5 indicate excellent performance. This metric is widely applied as a quality control standard to ensure reproducibility and reliability in large-scale compound screening.

### 2.4. Simulated Primary Screening Experiment Using the 96-Well Plate Assay

To validate the optimized antifungal screening protocol, a simulated primary screening experiment was conducted targeting *C. posadasii* spherules. A set of compounds with experimentally validated inhibitory activity against *C. posadasii* (NIC, NEN, and HEX) were selected. Duplicate “mock” screening library plates were prepared by spotting 1 µL of 1 mM stock drug solutions in DMSO into assigned wells of 96-well plates. Positive control wells contained amphotericin B (AmB) at a final concentration of 10 µg/mL, while negative and background controls included 1% DMSO and RPMI medium, respectively.

Briefly, a working inoculum of *C. posadasii* spherules (1 × 10^6^ spherules/mL) in RPMI medium was prepared as described. Each well of the mock screening drug plates was seeded to a final volume of 100 µL and incubated at 39 °C with 10% CO_2_ for 24 h. At the endpoint, 100 µL of XTT solution (0.5 mg/mL, supplemented with 40 µM menadione) was added to each well, followed by an additional 24 h incubation under the same conditions. Absorbance at 490 nm was measured using a microplate reader. Raw absorbance values were normalized to calculate the percentage of inhibition, using positive and negative controls for reference. To standardize the results, percentage inhibition values exceeding 100% or falling below 0% were adjusted to 100% and 0%, respectively.

### 2.5. Dose–Response Experiments for Confirmation of Screening Results Using the 96-Well Plate Assay

To confirm the inhibitory activity of potential hit compounds identified during the simulated primary screening, a secondary screening experiment was conducted using a typical dose–response assay. Briefly, serial two-fold dilutions of NIC, NEN, HEX, and AmB were prepared in 96-well plates in 50 µL of RPMI medium, with concentrations ranging from 40 µM to 0.0005 µM. Each well was then seeded with 50 µL of a working spherule inoculum (2 × 10^6^ spherules/mL), halving the compound concentrations. Plate incubation and XTT assays were conducted using the same conditions described in the screening assay above. OD_490_ values were measured using a microplate reader. As described previously, the percentage of growth inhibition was calculated and normalized relative to the positive (AmB) and negative (DMSO) controls for inhibition. Afterward, the inhibitory concentration required to reduce metabolic activity by 50% (IC_50_) was determined by fitting the normalized data to the variable slope Hill equation using GraphPad Prism (version v10.4.1). Dose–response assays were performed in duplicate, with three technical replicates per treatment at each concentration.

### 2.6. Growth of C. posadasii in 384-Well Plates for Assay Miniaturization, Optimization and Adaptation for Drug Screening

To miniaturize the multi-well plate protocol for use in a 384-well plate format, optimal conditions for the different parameters were extrapolated and adapted from our initial experiments using the lower-density 96-well plates. Briefly, a spherule working inoculum was prepared in RPMI medium (1 × 10^6^ spherules/mL), and 50 µL was dispensed into each well of the 384-well plates (Corning). Wells containing AmB (10 µg/mL) served as positive controls, while wells with non-drug media were used as negative controls. Following seeding, plates were incubated at 39 °C with 10% CO_2_ for 24 h. At the endpoint, 30 µL of XTT solution (0.5 mg/mL, supplemented with 40 µM menadione) was added to each well, and plates were further incubated under the same conditions. XTT-reduction was monitored at the indicated time points by measuring absorbance at 490 nm using a microplate reader. Absorbance readings were subsequently used to calculate the Z′ score statistics, as previously described [27].

### 2.7. Simulated Primary Screening Using the 384-Well Plate Assay

Once optimal conditions for the 384-well plate assay were determined, a simulated primary screening was performed by preparing “mock” screening library plates containing NIC, NEN, and HEX. Mock screening library plates were prepared in duplicate by spotting 1 µL of 0.5 mM stock drug solutions in DMSO into assigned wells of 384-well plates. Positive control wells contained AmB at a final concentration of 10 µg/mL, while negative and background controls included DMSO and RPMI medium, respectively. Plates were then seeded with a working inoculum of *C. posadasii* spherules (1 × 10^6^ spherules/mL) in RPMI medium, reaching a final volume of 50 μL per well. Plates were then incubated at 39 °C with 10% CO_2_ for 24 h. After the incubation, 30 µL of XTT solution (0.5 mg/mL, supplemented with 40 µM menadione) was added to each well, followed by an additional 24 h incubation. OD_490_ was monitored, and values were normalized to calculate the percentage of inhibition using positive and negative controls for reference.

### 2.8. Dose–Response Experiments for Confirmation of Screening Results Using the 384-Well Plate Assay

After identifying “potential hit” compounds from the mock screening library, dose–response assays were conducted to characterize their antifungal activity further. Serial two-fold dilutions of NIC, NEN, HEX, and AmB were prepared in 25 µL of RPMI. Each well was then seeded with 25 µL of a spherule working inoculum, resulting in final drug concentrations ranging from 20 µM to 0.0005 µM. Plates were incubated at 39 °C with 10% CO_2_ for 24 h. Afterward, 30 µL of XTT solution (0.5 mg/mL, supplemented with 40 µM menadione) was added to each well, and plates were further incubated under the same conditions. OD_490_ values were obtained, and IC_50_ values were determined by fitting the normalized OD_490_ data into the variable slope Hill equation using GraphPad Prism. Experiments were performed in duplicate, with three technical replicates per treatment.

## 3. Results

### 3.1. Optimization of an XTT-Based 96-Well Plate Protocol for the Growth of C. posadasii Spherules and to Identify Potential Active Antifungal Compounds

As previously described, due to the clinical significance of spherule initials during lung infection [28], we focused on optimizing an XTT-based antifungal assay, previously developed in our laboratory [29], with the main goal to simplify the experimental workflow. We first evaluated various experimental parameters in a preliminary set of experiments, including spherule inoculum density, menadione concentrations, and XTT-reduction incubation time (Supplementary Figure S1A). This initial approach identified optimal conditions that resulted in maximized Z′ scores, a critical statistic for ensuring reproducibility and robustness in large-scale screening assays [30].

**Figure 1:**
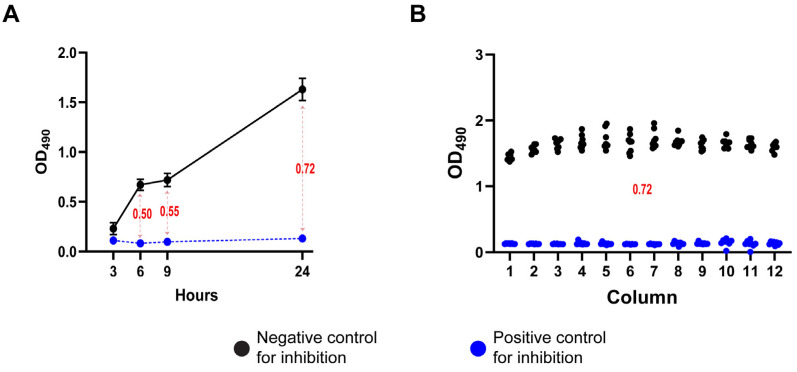
Optimization of experimental conditions for the 96-well plate assay. (**A**) Monitoring of the optimal incubation time for XTT reduction yielding the highest Z′ value; the blue line represents the OD_490_ readings from the positive control plate (Amb 10 µg/mL), the dark line represents the readings from the negative control plate (DMSO). (**B**) Representative Z′ assay using a positive and a negative control plate. Blue points represent one well of the positive control plate, while black points represent one of the negative control for inhibition. Numerical values in red indicate the calculated Z′ score at each time point, based on the corresponding control values.

We further demonstrated the robustness and reproducibility of the XTT-based metabolic assay using the effective antifungal AmB as a positive coccidioidal growth inhibition control. As illustrated in Figure 1A, the optimal assay conditions were determined to include a spherule inoculum of 1 × 10^6^ spherules/mL, an XTT solution (0.5 mg/mL) supplemented with 40 µM menadione, and a 24 h incubation period for XTT reduction. These conditions consistently yielded Z′ values exceeding 0.6, indicative of a highly reliable assay. The uniformity of the XTT signal across the assay plates further confirmed the reproducibility of the protocol (Figure 1B). Data are representative of experiments performed on at least three independent days (Supplementary Figure S2A).

### 3.2. Active Inhibitory Compounds on Spherules Were Correctly Identified in the 96-Well Plate Assay

**Figure 2:**
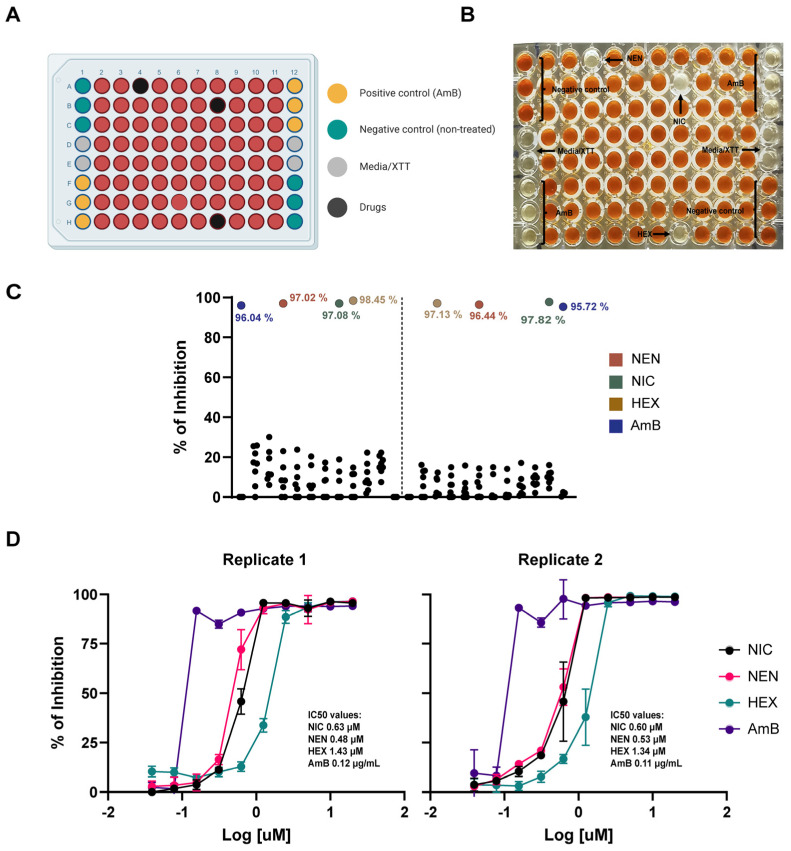
(**A**) Schematic view of a plate layout for a simulated primary screening experiment in a 96-well plate format. Wells in green represent the negative control for inhibition (untreated spherules), while wells in yellow correspond to the positive control (AmB). Gray wells indicate background controls, black wells represent drug-treated spherules, and red wells denote non-inhibition conditions (untreated, with XTT reduction). (**B**) Visual inspection of an XTT-based 96-well plate assay for drug screening. (**C**) Graphical representation of % of inhibition data from the “mock” primary screening performed in duplicate plates. Colored dots represent the percentage of inhibition of active drugs, while black dots represent non-inhibition wells. (**D**) Dose–response curves for each compound identified during the mock primary screening. IC_50_ values are calculated from concentration-dependent confirmatory experiments. Error bars represent standard deviations.

In the search for novel antifungals, chemical compound libraries are a valuable source of potentially active antifungal molecules [31,32]. Therefore, as proof of concept, we simulated a typical drug screening campaign using experimentally validated anti-coccidioidal compounds. We prepared “mock” screening libraries with AmB as the positive control to determine if our protocol could identify active compounds against *C. posadasii* spherules. Three compounds (NEN, NIC, and HEX) were selected for inclusion in the mock libraries based on their previously demonstrated activity, as identified in a screening campaign conducted earlier by our group [29]. The compounds were arrayed in a single plate, and a screening experiment was conducted in duplicate. Figure 2A shows the corresponding plate map. Visual inspection of the results (Figure 2B) confirmed the high activity of all three compounds. Furthermore, their percentage of inhibition was quantitatively validated in a duplicated experiment (Figure 2C).

### 3.3. Dose–Response Experiments Further Confirmed the Inhibitory Activity of Compounds on Spherules Using the 96-Well Plate Assay

As an additional validation step, we performed dose–response experiments to confirm the activity of the identified hits and determine their potency. As shown in Figure 2D, the IC_50_ values for the compounds were consistent across duplicate experiments.

### 3.4. Optimization of a Miniaturized 384-Well Plate Assay for High-Throughput Screening

Our subsequent experimental efforts focused on miniaturizing the protocol to a 384-well plate format. In large-scale drug screening campaigns, where thousands of compounds are evaluated for antifungal activity, optimizing assays to minimize material and reagent consumption, streamline experimental steps, reduce time, and lower costs are essential. Initially, the assay was optimized in a 96-well plate format, after which the experimental conditions were adapted to a 384-well plate format. To achieve this, we employed the same optimized conditions previously established for the 96-well plate assay. Additionally, we explored other alternative experimental conditions; however, these did not result in improved Z′ values. In its final format, the optimized conditions are spherule seeding with 50 µL of 1 × 10^6^ spherules/mL per well, an XTT solution with an initial menadione concentration of 40 µM, and an XTT-reduction time of 24 h.

**Figure 3:**
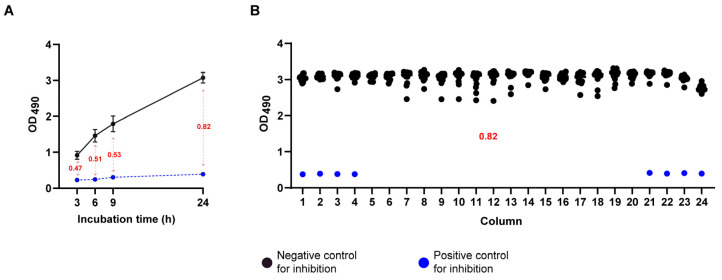
Miniaturization of an XTT-bases assay into a 384-well plate format. (**A**) Monitoring of the optimal incubation time for XTT reduction yielding the highest Z′ value; the Blue line represents the OD_490_ readings from the positive control for inhibition plate (AmB 10 µg/mL), the dark line represents the readings from the negative control for inhibition plate (DMSO). (**B**) Representative Z′ assay using a positive and a negative control for inhibition in a 384-well plate. Numerical values in red indicate the calculated Z′ score at each time point, based on the corresponding control values.

Consistent with the results obtained from the 96-well plate assay, we observed that a 24 h incubation enhanced XTT-reduction, resulting in Z′ values exceeding 0.7 (Figure 3A). Additionally, the uniformity of signals from both positive and negative controls across the plate was confirmed, further validating the robustness of the assay (Figure 3B). Data are representative of experiments performed on at least three independent days (Supplementary Figure S2B).

### 3.5. The Newly Developed and Optimized Miniaturized 384-Well Plate Assay Accurately Identified Tested Inhibitory Compounds

**Figure 4:**
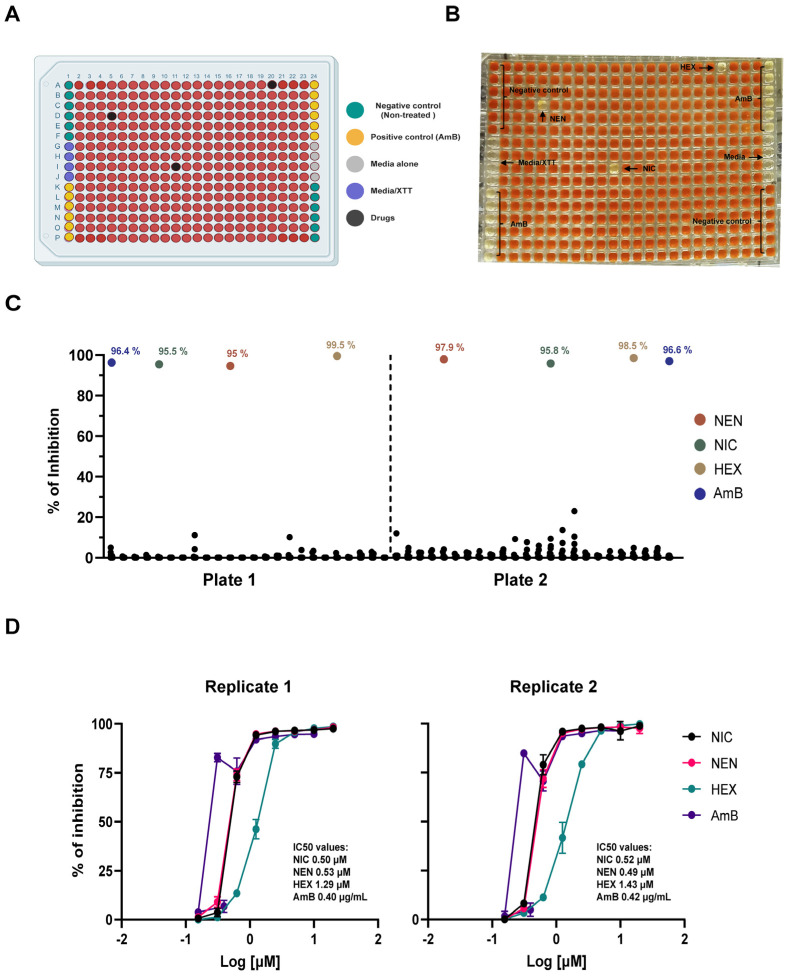
(**A**) Schematic view of a plate layout for miniaturized XTT-based 384-well plate format. Wells in green represent the negative control for inhibition (untreated spherules), while wells in yellow correspond to the positive control (AmB). Gray and purple wells indicate background controls, black wells represent drug-treated spherules, and red wells denote non-inhibition conditions (untreated, with XTT reduction). (**B**) Visual inspection for a simulated primary drug screening using an XTT-based 384-well plate format. (**C**) Graphical representation of % of inhibition data from the “mock” primary screening performed in duplicate plates. Colored dots represent the percentage of inhibition of active drugs, while black dots represent non-inhibition wells. (**D**) Dose–response curves for each compound identified during the mock primary screening. IC_50_ values are calculated from concentration-dependent confirmatory experiments. Error bars represent standard deviations.

Similarly, we simulated a primary drug screening campaign using the same compounds previously tested in the 96-well plate protocol. In the miniaturized 384-well plate format (Figure 4A), the compounds were successfully identified, even through visual inspection (Figure 4B), and their percentage inhibition was confirmed in duplicate plates (Figure 4C).

### 3.6. Dose–Response Experiments Using the 384-Well Plate Assay

Subsequently, we performed dose–response assays to validate the activity of the compounds and calculated their respective IC_50_ values (Figure 4D). Notably, the IC_50_ values obtained in the 384-well format were highly consistent with those from the 96-well assay (compare Figure 2D and Figure 4D).

## 4. Discussion

The primary antifungal agents for coccidioidomycosis, such as azoles and polyenes, are limited in their effectiveness and often have significant limitations that hinder long-term use. These limitations include mainly drug toxicity, adverse side effects, and the potential for developing resistance [33,34]. These challenges underscore the need to discover and develop novel antifungal agents with reduced toxicity and improved therapeutic profiles [35,36]. One of the main strategies to overcome such challenges is using large-scale screening, including high-throughput screening technologies [37]. For instance, by systematically applying HTS technology, it is possible to evaluate hundreds to millions of compounds for specific antifungal activity [38]. Moreover, we can experimentally narrow the chemical space for those exhibiting specific desired properties by sourcing these compounds from specialized libraries [39]. For example, it is possible to screen molecules from different libraries, such as FDA-approved drugs, de novo compounds with drug-like properties, diffusible blood–brain barrier molecules, etc.

Despite their widespread application in antimicrobial drug discovery, including antifungals, their use for targeting *Coccidioides* spp. remains limited. A significant barrier to their implementation for *Coccidioides* spp. is the requirement for Biosafety Level 3 (BSL-3) facilities, which are necessary to handle these pathogens safely [21]. This requirement introduces considerable logistical and operational complexities for screening under high containment levels, including stringent regulatory oversight, specialized infrastructure, and increased costs, hindering their broader application in coccidioidomycosis research and limiting the discovery of new antifungal agents for this neglected disease [23]. To our knowledge, there have been limited antifungal large-scale screening efforts against *Coccidioides* spp. Recently, Mead et al. reported screening the 1280-compound LOPAC library, identifying active inhibitory compounds on *Coccidioides* arthroconidia [40]. The screening protocol relied on measuring OD_600_ over 120 h, with fungal growth being the primary readout. Most recently, our group reported on the screening of compound libraries in search of potential antifungals that are effective against *C. posadasii* spherule initials [29]. The screening used an XTT-colorimetric technique to assess the metabolic activity of fungal elements in 96-well microtiter plates, identifying 254 potential drugs that inhibited more than 70% of the metabolism of the cells. However, the associated methodology was relatively cumbersome, performed under BSL-3 conditions, and consisted of many steps that significantly limited the utility and overall throughput of these screening techniques. At the same time, we also reported on the possibility of using an attenuated mutant strain of *C. posadasii* (C735 *∆cts2/∆ard1/∆cts3*, or *∆T*) as a surrogate for the wild-type BSL-3 strain in this type of screening assays [23]. Thus, we posited that this attenuated strain, which can be handled under BSL-2 conditions, constitutes an ideal tool allowing for the manipulation of different experimental parameters for the optimization of the screening methodologies, thereby potentially circumventing the limitations associated with working in high containment (BSL-3) environments.

As our main target for drug discovery is spherule initials, we decided to use the existing XTT protocol described recently by our group as a starting point. Therefore, we modified key parameters to suit our experimental setup, aiming to streamline the experimental workflow and increase the throughput of potential screening efforts. To establish a robust and reproducible high-throughput screening assay, we evaluated different experimental parameters, including initial inoculum size, menadione concentration, and XTT-reduction time. By optimizing key parameters, our protocol increases the efficiency of spherule production and the throughput for screening thousands of compounds per experimental run. Of note, the experimental conditions initially established in this study address a potential limitation associated with producing sufficient spherule numbers in vitro. Spherules derived from arthroconidia only enlarge in cell size without cell division over 5–7 days. Furthermore, a mature spherule at 5–7 days post inoculation can produce 300–800 endospores, which form clusters, making it challenging to normalize cell numbers for this assay [41]. It is reasonable to use spherule initials and mature spherules before endosporulation for these screening assays; however, preparing a large quantity of spherules is a critical limiting step for these assays. Thus, compared to our original report [29] our optimized protocol uses 1/10th of the concentration of cells and ½ the volume in the initial inoculum per well. Thus, by itself, this has the potential to increase the throughput already by a factor of 20, even when using the lower-density 96-well microtiter plate format. Additionally, fulfilling one of our initial objectives, the optimized protocol also minimizes the number of experimental steps, particularly those that are more labor-intensive and time-consuming (i.e., washings, centrifugation, filtration, etc.) and thereby more prone to introducing operator-related error, which could potentially compromise the reproducibility of the technique [42]. Overall, we found that the best conditions were using a working inoculum of 1 × 10^6^ spherules/mL, an initial menadione concentration of 40 µM, and an XTT-reduction time of 24 h. Using the optimal experimental conditions, we consistently obtained Z′ score values exceeding 0.6 across multiple plates and experimental days for both well-plate formats. It is particularly crucial for primary screening at single-compound concentrations, as this step depends on high Z′ values to identify active compounds that warrant further evaluation through dose–response analysis [30]. It is also important to note that this protocol can be readily adapted to other BSL-2 and BSL-3 *Coccidioides* strains, although minor experimental adjustments may be required to optimize conditions for each specific strain.

To further validate the developed protocols, we performed a primary mock screening to assess whether potential hits could be accurately identified using our assay. The compounds chosen for this screening were a subset previously known to show activity against *Coccidioides* spherules [29]. Using both 96-well and 384-well plate formats, we successfully identified three active “hit” compounds. These hits displayed consistent inhibition percentages across replicates in both formats, demonstrating the robustness of the protocols. Moreover, the inhibition values were similar between the two plate formats, highlighting their interchangeability and adaptability for laboratories with varying equipment. Finally, we conducted follow-up experiments to simulate a typical secondary confirmation screening. Through dose–response assays, we calculated the IC_50_ values for NIC, NEN, HEX, and AmB, which proved to be highly reproducible across different days. Furthermore, the IC_50_ values were comparable between the 96-well and 384-well plate formats, further validating the reliability and repeatability of the protocols.

Overall, in this study, we established and optimized robust protocols for large-scale screening, including “true” high-throughput screening (HTS), enabling the safe and accessible evaluation of compound libraries against *Coccidioides* spherule initials. To enhance accessibility and scalability, we adapted the protocol into both 96-well and 384-well plate formats, ensuring compatibility with a wide range of laboratory setups. Furthermore, by using the attenuated *C. posadasii* C735 *∆cts2/∆ard1/∆cts3 (∆T)* strain, our protocol enables drug screening campaigns to be performed under BSL-2 facilities. This advancement not only accelerates the identification of novel antifungal agents for coccidioidomycosis but also provides a scalable framework for future research on this and other high-containment pathogens.

## Data Availability

No new data were created or analyzed in this study. Data sharing is not applicable to this article.

## Supplementary Materials

The following supporting information can be downloaded at: https://www.mdpi.com/article/10.3390/jof11100733/s1, Figure S1. Optimization of experimental conditions for an XTT-based 96-well plate assay for antifungal drug screening on *C. posadasii*; Figure S2. Evaluation of the reproducibility and assay quality of the XTT-based well plate assay for antifungal drug screening on *Coccidiodes* spherules.

## References

[1] Crum N.F. (2022). Coccidioidomycosis: A contemporary review. Infect. Dis. Ther..

[2] Thompson G.R., Chiller T.M. (2022). Endemic Mycoses: Underdiagnosed and Underreported.

[3] Kirkland T.N., Stevens D.A., Hung C.Y., Beyhan S., Taylor J.W., Shubitz L.F., Duttke S.H., Heidari A., Johnson R.H., Deresinski S.C. (2022). Coccidioides Species: A Review of Basic Research: 2022. J. Fungi.

[4] Donovan F.M., Fernández O.M., Bains G., DiPompo L. (2025). Coccidioidomycosis: A growing global concern. J. Antimicrob. Chemother..

[5] Koutserimpas C., Naoum S., Melissinos E.P., Raptis K., Alpantaki K., Dretakis K., Piagkou M., Samonis G. (2023). Spinal Infections Caused by Coccidioides Species. Maedica.

[6] Koutserimpas C., Naoum S., Raptis K., Vrioni G., Samonis G., Alpantaki K. (2022). Skeletal infections caused by Coccidioides species. Diagnostics.

[7] Moni B.M., Wise B.L., Loots G.G., Weilhammer D.R. (2023). Coccidioidomycosis osteoarticular dissemination. J. Fungi.

[8] Benedict K. (2019). Surveillance for coccidioidomycosis—United States, 2011–2017. MMWR Surveill. Summ..

[9] Centers for Disease Control and Prevention (2024). Valley Fever (Coccidioidomycosis) Statistics. https://www.cdc.gov/valley-fever/php/statistics/index.html.

[10] World Health Organization (2022). WHO Fungal Priority Pathogens List to Guide Research, Development and Public Health Action.

[11] Tverdek F.P., Kofteridis D., Kontoyiannis D.P. (2016). Antifungal agents and liver toxicity: A complex interaction. Expert Rev. Anti-Infect. Ther..

[12] Allen U.D., Canadian Paediatric Society and Infectious Diseases and Immunization Committee (2010). Antifungal agents for the treatment of systemic fungal infections in children. Paediatr. Child Health.

[13] Souza A.C., Amaral A.C. (2017). Antifungal therapy for systemic mycosis and the nanobiotechnology era: Improving efficacy, biodistribution and toxicity. Front. Microbiol..

[14] Broach J.R., Thorner J. (1996). High-throughput screening for drug discovery. Nature.

[15] Martis E., Radhakrishnan R., Badve R. (2011). High-throughput screening: The hits and leads of drug discovery-an overview. J. Appl. Pharm. Sci..

[16] Ayon N.J. (2023). High-throughput screening of natural product and synthetic molecule libraries for antibacterial drug discovery. Metabolites.

[17] Farha M.A., Brown E.D. (2019). Drug repurposing for antimicrobial discovery. Nat. Microbiol..

[18] Aggarwal M., Patra A., Awasthi I., George A., Gagneja S., Gupta V., Capalash N., Sharma P. (2024). Drug repurposing against antibiotic resistant bacterial pathogens. Eur. J. Med. Chem..

[19] Cheng Y.-S., Williamson P.R., Zheng W. (2019). Improving therapy of severe infections through drug repurposing of synergistic combinations. Curr. Opin. Pharmacol..

[20] Vazquez-Rodriguez A., Rodrigues C.F., Ramage G., Lopez-Ribot J.L., Ramage G., Andes D. (2025). Large-Scale Phenotypic Screenings of Repurposing Libraries to Identify Drugs with Novel Antifungal Activity Against Candida Biofilms. Fungal Biofilms.

[21] Garcia J.A., Vu K., Thompson G.R., Gelli A. (2022). Characterization of the growth and morphology of a BSL-2 Coccidioides posadasii strain that persists in the parasitic life cycle at ambient CO_2_. J. Fungi.

[22] Mead H.L., Blackmon A.V., Vogler A.J., Barker B.M. (2019). Heat Inactivation of Coccidioides posadasii and Coccidioides immitis for use in lower biosafety containment. Appl. Biosaf..

[23] Xue J., Chen X., Selby D., Hung C.Y., Yu J.J., Cole G.T. (2009). A genetically engineered live attenuated vaccine of Coccidioides posadasii protects BALB/c mice against coccidioidomycosis. Infect. Immun..

[24] Converse J.L., Besemer A.R. (1959). Nutrition of the parasitic phase of Coccidioides immitis in a chemically defined liquid medium. J. Bacteriol..

[25] Zhang J.-H., Chung T.D., Oldenburg K.R. (1999). A simple statistical parameter for use in evaluation and validation of high throughput screening assays. J. Biomol. Screen..

[26] Pierce C.G., Uppuluri P., Tristan A.R., Wormley F.L., Mowat E., Ramage G., Lopez-Ribot J.L. (2008). A simple and reproducible 96-well plate-based method for the formation of fungal biofilms and its application to antifungal susceptibility testing. Nat. Protoc..

[27] Zhang J.-H., Oldenburg K.R., Schwab M. (2017). Z-Factor. Encyclopedia of Cancer.

[28] Sun S.H., Cole G.T., Drutz D.J., Harrison J.L. (1986). Electron-microscopic observations of the Coccidioides immitis parasitic cycle in vivo. J. Med. Vet. Mycol..

[29] Saeger S., West-Jeppson K., Liao Y.R., Campuzano A., Yu J.J., Lopez-Ribot J., Hung C.Y. (2025). Discovery of novel antifungal drugs via screening repurposing libraries against *Coccidioides posadasii* spherule initials. mBio.

[30] Coma I., Herranz J., Martin J. (2009). Statistics and Decision Making in High-Throughput Screening. High Throughput Screening: Methods and Protocols.

[31] Ajetunmobi O.H., Chaturvedi A.K., Badali H., Vaccaro A., Najvar L., Wormley F.L., Wiederhold N.P., Patterson T.F., Lopez-Ribot J.L. (2023). Screening the medicine for malaria venture’s Pandemic Response Box to identify novel inhibitors of Candida albicans and Candida auris biofilm formation. APMIS.

[32] Ajetunmobi O.H., Wall G., Vidal Bonifacio B., Martinez Delgado L.A., Chaturvedi A.K., Najvar L.K., Wormley F.L., Patterson H.P., Wiederhold N.P., Patterson T.F. (2023). High-throughput screening of the repurposing hub library to identify drugs with novel inhibitory activity against Candida albicans and Candida auris biofilms. J. Fungi.

[33] Goughenour K.D., Rappleye C.A. (2017). Antifungal therapeutics for dimorphic fungal pathogens. Virulence.

[34] Thompson G.R., Barker B.M., Wiederhold N.P. (2017). Large-scale evaluation of in vitro amphotericin B, triazole, and echinocandin activity against Coccidioides species from US institutions. Antimicrob. Agents Chemother..

[35] Zhou Z.X., Yin X.D., Zhang Y., Shao Q.H., Mao X.Y., Hu W.J., Shen Y.L., Zhao B., Li Z.L. (2022). Antifungal Drugs and Drug-Induced Liver Injury: A Real-World Study Leveraging the FDA Adverse Event Reporting System Database. Front. Pharmacol..

[36] Yang Y.L., Xiang Z.J., Yang J.H., Wang W.J., Xu Z.C., Xiang R.L. (2021). Adverse Effects Associated With Currently Commonly Used Antifungal Agents: A Network Meta-Analysis and Systematic Review. Front. Pharmacol..

[37] Patel C.N., Shakeel A., Mall R., Alawi K.M., Ozerov I.V., Zhavoronkov A., Castiglione F. (2025). Strategies for Redesigning Withdrawn Drugs to Enhance Therapeutic Efficacy and Safety: A Review. Wiley Interdiscip. Rev. Comput. Mol. Sci..

[38] Wall G., Lopez-Ribot J.L. (2020). Screening repurposing libraries for identification of drugs with novel antifungal activity. Antimicrob. Agents Chemother..

[39] Olmedo D.A., Durant-Archibold A.A., López-Pérez J.L., Medina-Franco J.L. (2024). Design and Diversity analysis of chemical libraries in drug discovery. Comb. Chem. High Throughput Screen..

[40] Mead H.L., Valentine M., Yin H., Thompson G.R., Keim P., Engelthaler D.M., Barker B.M. (2024). In vitro small molecule screening to inform novel candidates for use in fluconazole combination therapy in vivo against Coccidioides. Microbiol. Spectr..

[41] Mead H.L., Teixeira M.D.M., Galgiani J.N., Barker B.M. (2018). Characterizing in vitro spherule morphogenesis of multiple strains of both species of Coccidioides. Med. Mycol..

[42] Macarrón R., Hertzberg R.P. (2011). Design and implementation of high throughput screening assays. Mol. Biotechnol..

