# Supplementary material for: Development and Optimization of Multi-Well Colorimetric Assays for Growth of Coccidioides posadasii Spherules and Their Application in Large-Scale Screening
Source: J Fungi (Basel). 2025 Oct 11;11(10):733. doi: 10.3390/jof11100733 (PMC12565162; doi:10.3390/jof11100733)
Supplement: Supplementary file 1 [file jof-11-00733-s001.zip › jof-3868110-supplementary.pdf]

Sup Fig 1

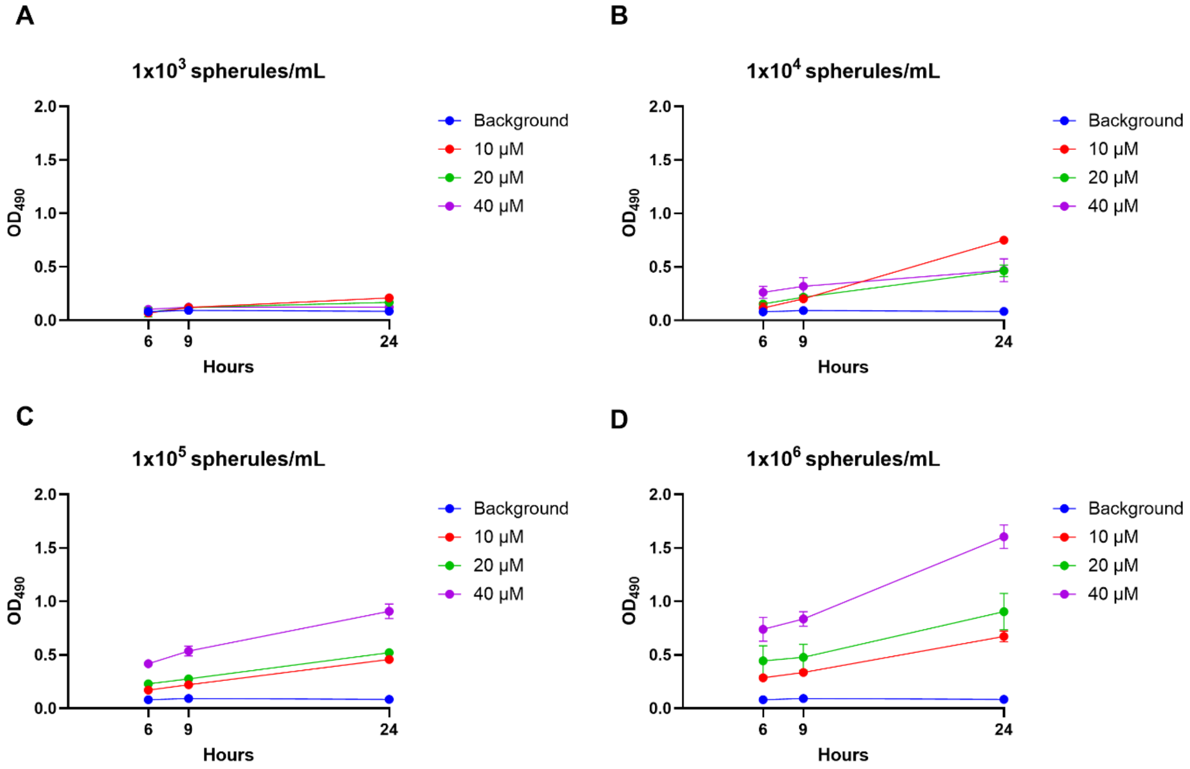

Supplementary Figure 1. Optimization of experimental conditions for an XTT-based 96-well plate assay for antifungal drug screening on *C. posadasii*. Different conditions were tested to obtain the highest separation band between the reduced-XTT signal between the background and cell controls. Initial inoculum in spherules/mL, XTT supplemented with different menadione concentrations (10. 20 and 40 µM), and XTT-reduction incubation time were analyzed.

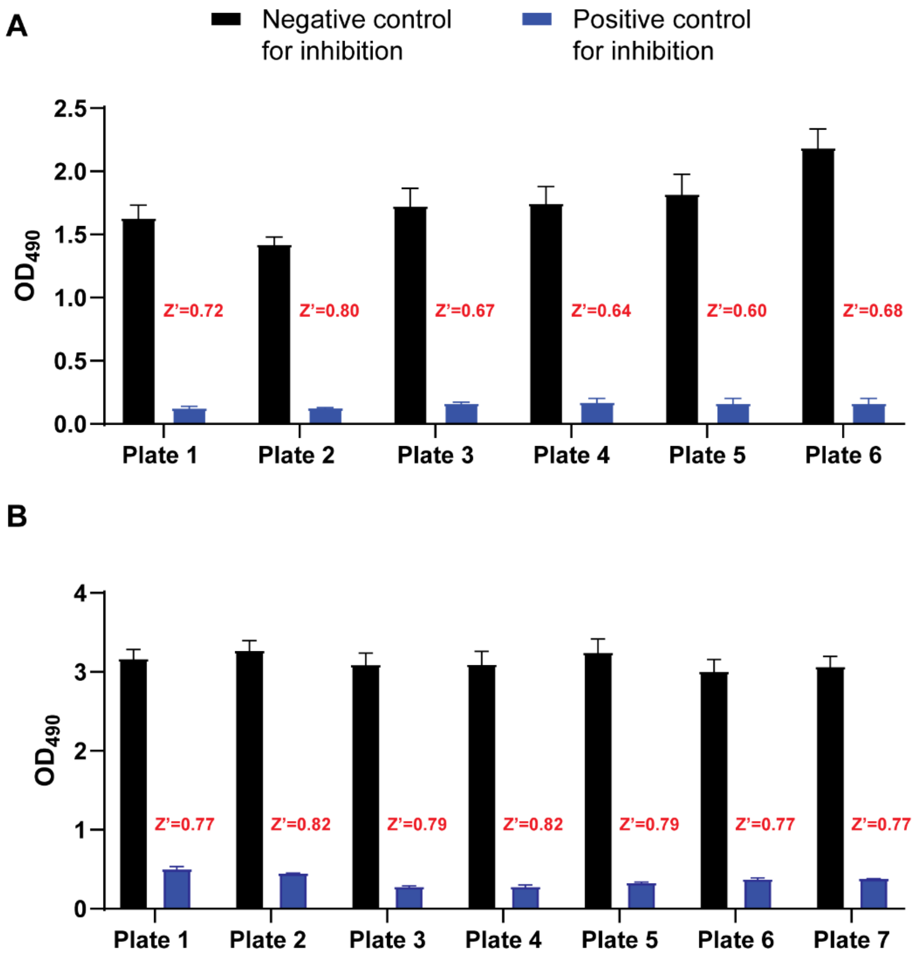

Supplementary Figure 2. Evaluation of the reproducibility and assay quality of the XTT-based well plate assay for antifungal drug screening on *Coccidiodes* spherules. **A**) Reproducibility of the XTT-based 96-well plate format assay. OD_{490} readings from positive control wells containing AmB at 10 µg/mL (blue bar) and negative control wells with no drug (black bar). **B**) Reproducibility of the XTT-based 384-well plate format assay. OD_{490} readings from positive control wells containing AmB at 10 µg/mL (blue bar) and negative control wells with no drug (black bar). For both plate formats, Z' values were calculated for each plate, exhibiting values higher than 0.6 for each case; experimental runs were performed in at least 3 independent days.
